# Atypical Hemorrhagic Presentation of Neurocysticercosis in a Patient on Chronic Anticoagulation

**DOI:** 10.7759/cureus.104456

**Published:** 2026-02-28

**Authors:** Eliany Leon Figueredo, Elizabeth Blanco Espinosa, Manuel A Guerra Guerrero, Ruben E Diaz Samada, Maria de la Caridad Morales Fernández, Dainiel Diaz Acosta

**Affiliations:** 1 Internal Medicine, Englewood Health Physician Network, New Jersey, USA; 2 General Medicine, Ceda Orthopedic Group, Miami, USA; 3 General Surgery, Hospital Universitario Arnaldo Milian Castro, Santa Clara, CUB; 4 Community Medicine, Community Medical Group, Lakeland, USA; 5 Internal Medicine, Saturnino Lora General Hospital, Santiago de Cuba, CUB; 6 Dermatology, University of Miami Hospital, Miami, USA; 7 General Medicine, Universidad de Ciencias Médicas de Cienfuegos, Cienfuegos, CUB

**Keywords:** anticoagulation, brain mri, intraparenchymal hemorrhage, neurocysticercosis, scolex, warfarin

## Abstract

Neurocysticercosis (NCC) is a common parasitic infection of the central nervous system in individuals from endemic regions, but hemorrhagic presentations are rare and may closely mimic neoplastic or vascular lesions, particularly in anticoagulated patients. We describe the case of a 68-year-old woman with a mechanical aortic valve on chronic warfarin therapy who presented with acute confusion and was found to have a left frontal intraparenchymal hemorrhage. Neuroimaging demonstrated a hemorrhagic cystic lesion with an eccentric intracystic nodule suggestive of a scolex, raising concern for NCC despite negative serologic testing. Because serologic assays have limited sensitivity in solitary parenchymal disease, the diagnosis was approached with uncertainty and relied on the characteristic MRI appearance, epidemiologic risk, and the lesion’s progressive radiologic regression on follow-up imaging. Management included reversal and temporary suspension of anticoagulation, initiation of albendazole with corticosteroids, seizure prophylaxis, and treatment of hyponatremia, partially pseudohyponatremic due to hyperglycemia but ultimately consistent with syndrome of inappropriate antidiuretic hormone secretion (SIADH). The patient showed gradual clinical improvement and near-complete radiologic resolution over several months. This case underscores the diagnostic challenges of hemorrhagic NCC, the limited sensitivity of serologic assays in solitary parenchymal lesions, and the potential for anticoagulation to amplify hemorrhagic complications. It also highlights the essential role of serial neuroimaging and multidisciplinary management in guiding therapy and avoiding unnecessary surgical intervention.

## Introduction

Neurocysticercosis (NCC), an infection caused by the larval stage of *Taenia solium*, remains the most common parasitic disease of the central nervous system and a leading cause of acquired epilepsy, particularly in regions such as Colombia, Peru, and other parts of Latin America, sub-Saharan Africa, and South Asia. Although NCC predominantly presents with seizures, headaches, or focal neurological deficits, its clinical and radiological spectrum is broad, and atypical manifestations may complicate timely diagnosis. One such uncommon manifestation is hemorrhagic NCC, a rare presentation that may mimic neoplastic, vascular, or inflammatory lesions, especially in older adults or in patients with comorbidities that predispose to bleeding [1].

The diagnostic evaluation of NCC is often challenging because no single test provides definitive confirmation in all settings. While neuroimaging has become central to diagnosis, classic radiologic features, such as a cystic lesion with an eccentric mural nodule representing the scolex, may be obscured in hemorrhagic or degenerating states. Furthermore, serologic testing has limited sensitivity in patients with solitary parenchymal lesions, leading to false-negative results that complicate interpretation [2]. This combination of atypical imaging findings and limited serologic accuracy often results in NCC being misdiagnosed as a primary brain tumor, metastasis, or vascular malformation, potentially prompting unnecessary surgical interventions [3].

The complexity increases in individuals receiving chronic anticoagulation, where intracranial hemorrhage may have multifactorial origins. Anticoagulants such as warfarin elevate the risk of bleeding from otherwise indolent lesions, blurring the distinction between spontaneous hemorrhage, hemorrhage secondary to infection, and hemorrhage from structural vascular abnormalities. Accordingly, the diagnostic process in such patients requires careful integration of radiologic evolution, epidemiologic risk factors, and clinical response to therapy [4].

This case report describes an elderly woman originally from an endemic region who presented with an acute intracerebral hemorrhage as the first manifestation of NCC while on chronic warfarin therapy for a mechanical aortic valve. It highlights the diagnostic uncertainty created by hemorrhagic lesions on imaging, the limited reliability of serologic testing, and the need for a multidisciplinary approach that balances anticoagulation management, infectious disease considerations, and neurocritical care. Furthermore, it underscores the importance of serial neuroimaging in guiding diagnosis and avoiding unnecessary invasive procedures.

## Case presentation

**Figure 1:**
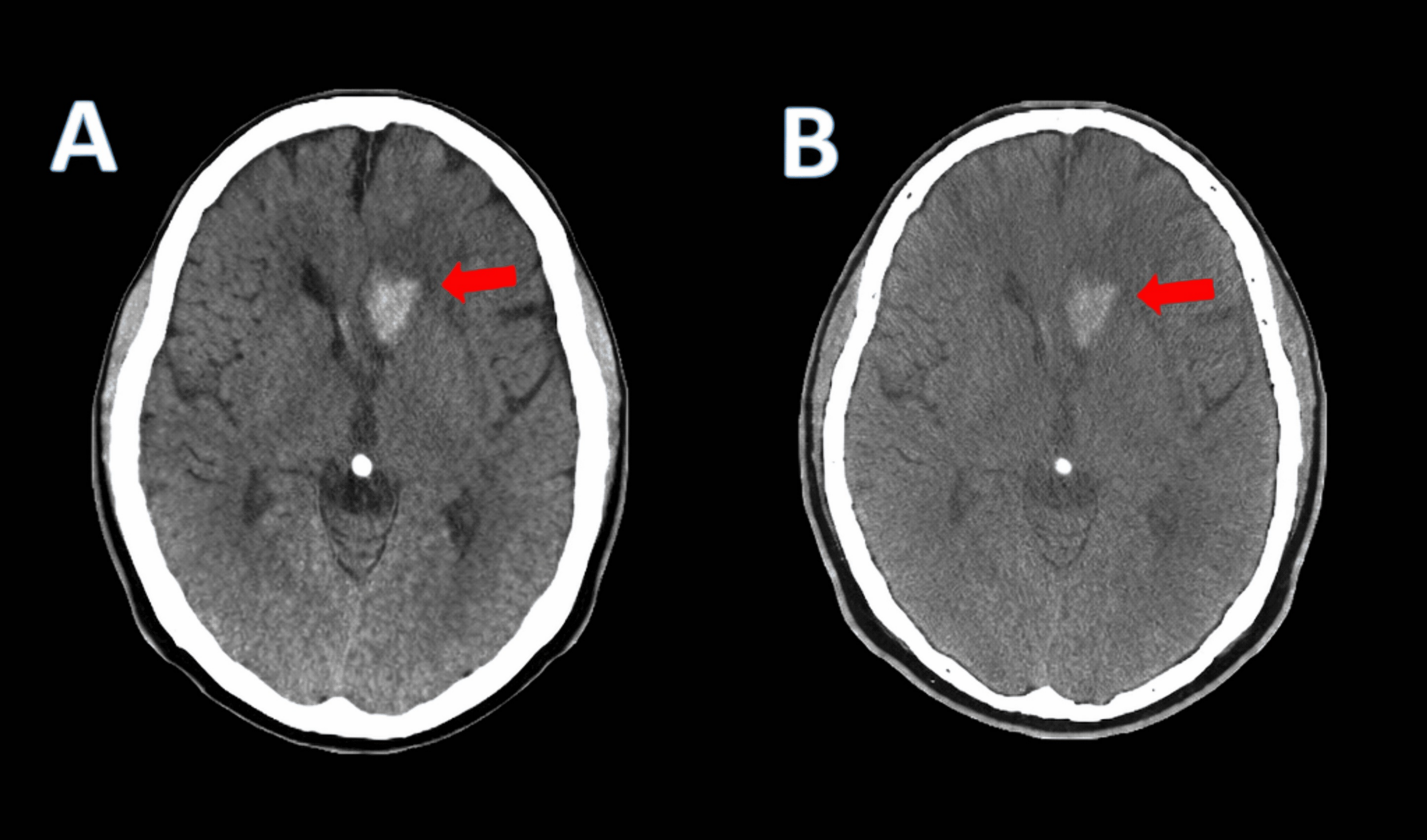
Non-contrast axial CT of the head. (A) Acute left frontal intraparenchymal hemorrhage with surrounding vasogenic edema (red arrow). (B) Adjacent intraventricular extension into the left frontal horn (red arrow).

A 68-year-old woman originally from Colombia, with a past medical history significant for mechanical aortic valve replacement on chronic warfarin therapy, type 2 diabetes mellitus, pulmonary hypertension, dyslipidemia, osteopenia, and remote colon cancer, presented to the emergency department with an acute onset of confusion, unsteady gait, and generalized weakness. On arrival, non-contrast CT of the head demonstrated an acute left frontal intraparenchymal hemorrhage measuring approximately 2.8 cm, with mild surrounding vasogenic edema and local mass effect, without midline shift. A small amount of adjacent intraventricular hemorrhage involving the left frontal horn and trace subarachnoid hemorrhage was also noted. No underlying mass lesion was clearly identified on CT (Figure 1).

Initial laboratory evaluation demonstrated significant electrolyte and osmolar abnormalities, including partial pseudohyponatremia due to marked hyperglycemia, as summarized in Table 1. After applying the standard correction formula for hyperglycemia, the patient’s sodium level increased from 120 mmol/L to 125 mmol/L, confirming that the hyponatremia was only partially artifactual and remained clinically significant. Hypertonic saline and fluid restriction were initiated, leading to gradual improvement in mental status. Despite the contribution of hyperglycemia, the persistently low corrected sodium, elevated urine osmolality, and elevated urine sodium remained consistent with syndrome of inappropriate antidiuretic hormone secretion (SIADH) physiology.

**Table 1:** Summary of initial laboratory findings. Na, sodium; K, potassium; INR, international normalized ratio; SIADH, syndrome of inappropriate antidiuretic hormone secretion

Test	Value	Reference Range	Interpretation
Na	120 mmol/L → 125 mmol/L (after correction)	135-145 mmol/L	Severe hyponatremia, consistent with SIADH
Glucose	>290 mg/dL	70-140 mg/dL	Marked hyperglycemia, contributing to pseudohyponatremia
K	3.3 mmol/L	3.5-5.1 mmol/L	Mild hypokalemia
Creatinine	Normal	0.6-1.3 mg/dL	Preserved renal function
INR	1.9	2.5-3.5	Subtherapeutic, but still increases hemorrhagic risk
Serum osmolality	Low	275-295 mOsm/kg	Supports SIADH
Urine osmolality	Inappropriately high	>100 mOsm/kg	Inappropriately concentrated, consistent with SIADH
Urine sodium	Elevated	>30 mmol/L	Consistent with SIADH physiology

**Figure 2:**
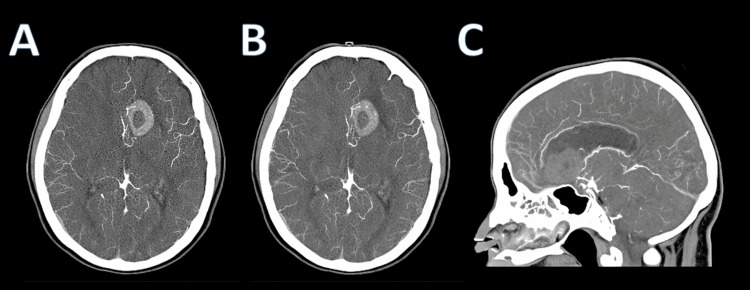
CTA of the head. (A, B) Axial CTA images showing the left frontal hemorrhagic lesion with adjacent vascular structures. (C) Sagittal CTA view demonstrating the vascular anatomy and absence of a focal malformation at the hemorrhage site. CTA, computed tomography angiography

Computed tomography angiography (CTA) of the head demonstrated a small 3-mm medially directed aneurysm at the origin of the left posterior communicating artery. Additionally, there was decreased caliber of the anterior communicating artery and proximal anterior cerebral artery segments, felt to be secondary to mass effect from the adjacent hemorrhage or reactive vasospasm. No vascular malformation was identified at the site of hemorrhage (Figure 2).

**Figure 3:**
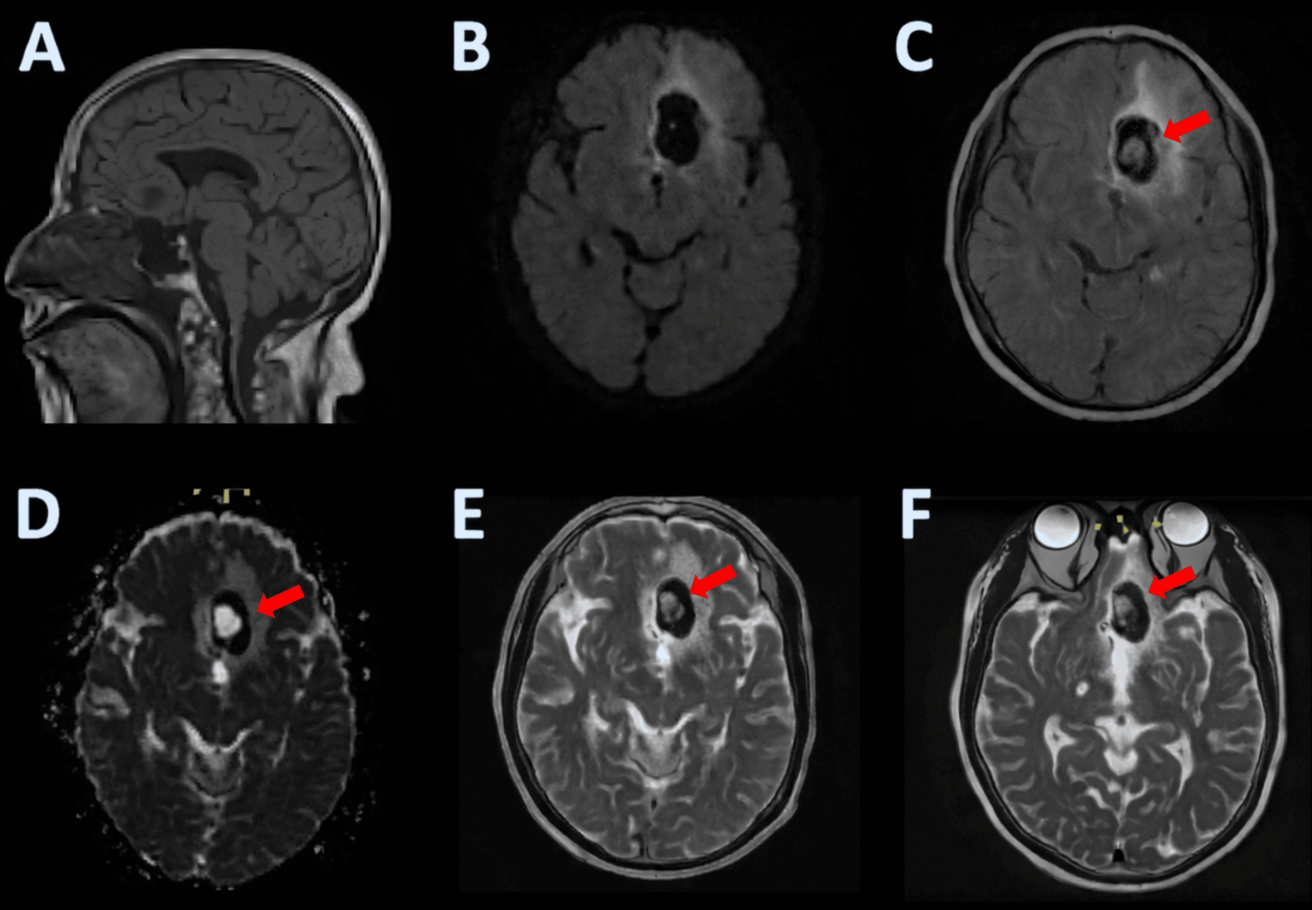
Brain MRI. (A) Sagittal T1-weighted image showing the left frontal hemorrhagic cystic lesion. (B) Axial DWI demonstrating restricted diffusion along the left cingulate region. (C) Axial FLAIR image highlighting the cystic lesion with surrounding edema; the red arrow marks the eccentric intracystic nodule suspicious for a scolex. (D) ADC map corresponding to areas of restricted diffusion, with the red arrow indicating the intracystic nodule. (E) Axial T2-weighted image demonstrating the internal hyperintense cystic component and the intracystic nodule (red arrow). (F) Axial T2-weighted image showing adjacent hemorrhagic changes and the intracystic nodule marked by a red arrow. DWI, diffusion-weighted imaging; ADC, apparent diffusion coefficient; FLAIR, fluid-attenuated inversion recovery

Subsequent MRI of the brain with and without contrast demonstrated a 3.0-cm hemorrhagic cystic mass in the left inferomedial frontal lobe, containing an internal T2-hyperintense cystic component with an intracystic nodular focus suspicious for a scolex. The lesion demonstrated minimal enhancement and was associated with surrounding hemorrhage, raising concern for NCC. Additionally, restricted diffusion was noted in the left cingulate gyrus, concerning for an acute ischemic infarct versus diffusion changes related to adjacent hemorrhage (Figure 3).

Given the patient’s history and the imaging characteristics, a broad differential diagnosis was considered. NCC was strongly suspected due to the cystic component with a nodular intracystic focus consistent with a possible scolex. However, several alternative etiologies, including a hemorrhagic primary brain tumor, metastatic disease in the context of her remote colon cancer, an atypical abscess, a cavernous malformation, and hemorrhage related to anticoagulation alone, remained plausible. The small posterior communicating artery aneurysm identified on CTA was evaluated as a potential bleeding source but was ultimately deemed incidental and unrelated. The constellation of findings prompted close monitoring and further evaluation to clarify the underlying etiology.

During hospitalization, serologic testing for *Taenia solium* using Western blot was performed and returned negative, highlighting that the diagnosis was primarily based on imaging findings and clinical context. Warfarin therapy was reversed with four-factor prothrombin complex concentrate (PCC) and subsequently held due to intracranial hemorrhage. Antiparasitic therapy with albendazole was initiated in combination with corticosteroids, along with levetiracetam for seizure prophylaxis. Ophthalmologic evaluation was recommended to assess for ocular involvement prior to and during antiparasitic treatment.

Regarding anticoagulation management, the patient’s initial international normalized ratio on admission was 1.9. Warfarin was immediately discontinued, and anticoagulation reversal was performed using PCC within the first hour of presentation. Given the acute intraparenchymal hemorrhage, pharmacologic bridging with heparin was not initiated, consistent with American Heart Association/American Stroke Association (AHA/ASA) guidelines for vitamin K antagonist-associated intracranial hemorrhage. Once radiographic stability was confirmed and the risk of expansion had decreased, the multidisciplinary team determined that anticoagulation could be cautiously resumed. Warfarin was restarted several days later without bridging therapy, balancing the high thromboembolic risk associated with the patient’s mechanical aortic valve against the need to minimize the risk of rebleeding. The patient demonstrated progressive clinical improvement and returned to her neurological baseline prior to discharge.

**Figure 4:**
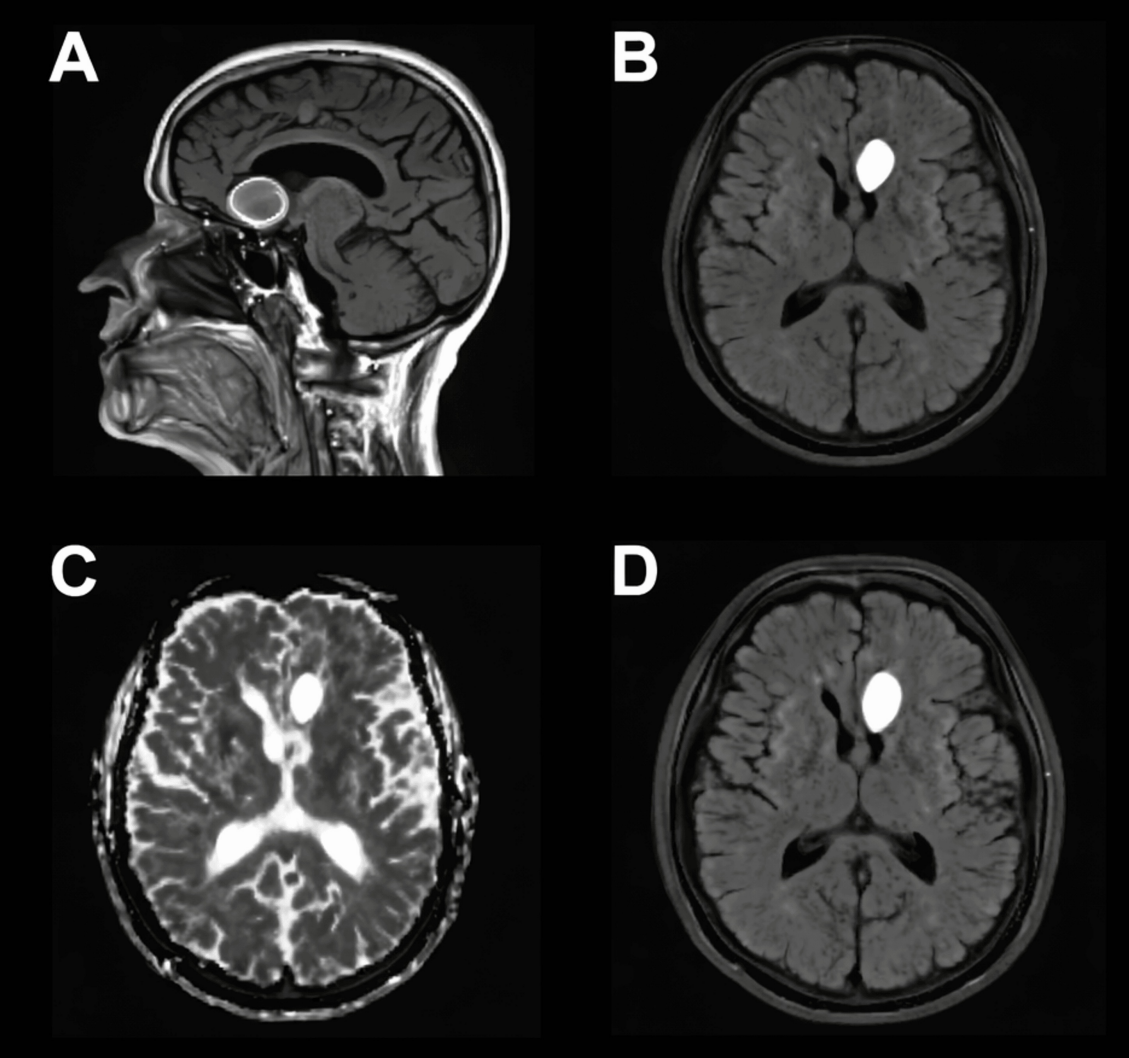
Follow-up brain MRI one month after initial presentation. A. Sagittal T1-weighted MRI demonstrating the intraparenchymal lesion in the left inferomedial frontal lobe. B. Axial FLAIR sequence showing the residual hyperintense cystic component within the lesion. C. Axial T2-weighted MRI highlighting internal cystic signal characteristics. D. Axial FLAIR MRI depicting interval reduction in lesion size with resolution of perilesional edema. FLAIR, fluid-attenuated inversion recovery

Follow-up MRI of the brain performed one month later demonstrated a decrease in the size of the left inferomedial frontal intraparenchymal hemorrhagic mass, now measuring approximately 2.9 × 1.8 × 2.5 cm compared to 3.0 × 2.1 × 3.0 cm on prior imaging. The lesion remained nonenhancing and contained a persistent internal T2-hyperintense cystic component, with complete resolution of the surrounding perilesional edema. These findings favored an evolving inflammatory or infectious process, such as NCC, although continued follow-up was recommended to exclude alternative etiologies (Figure 4).

**Figure 5:**
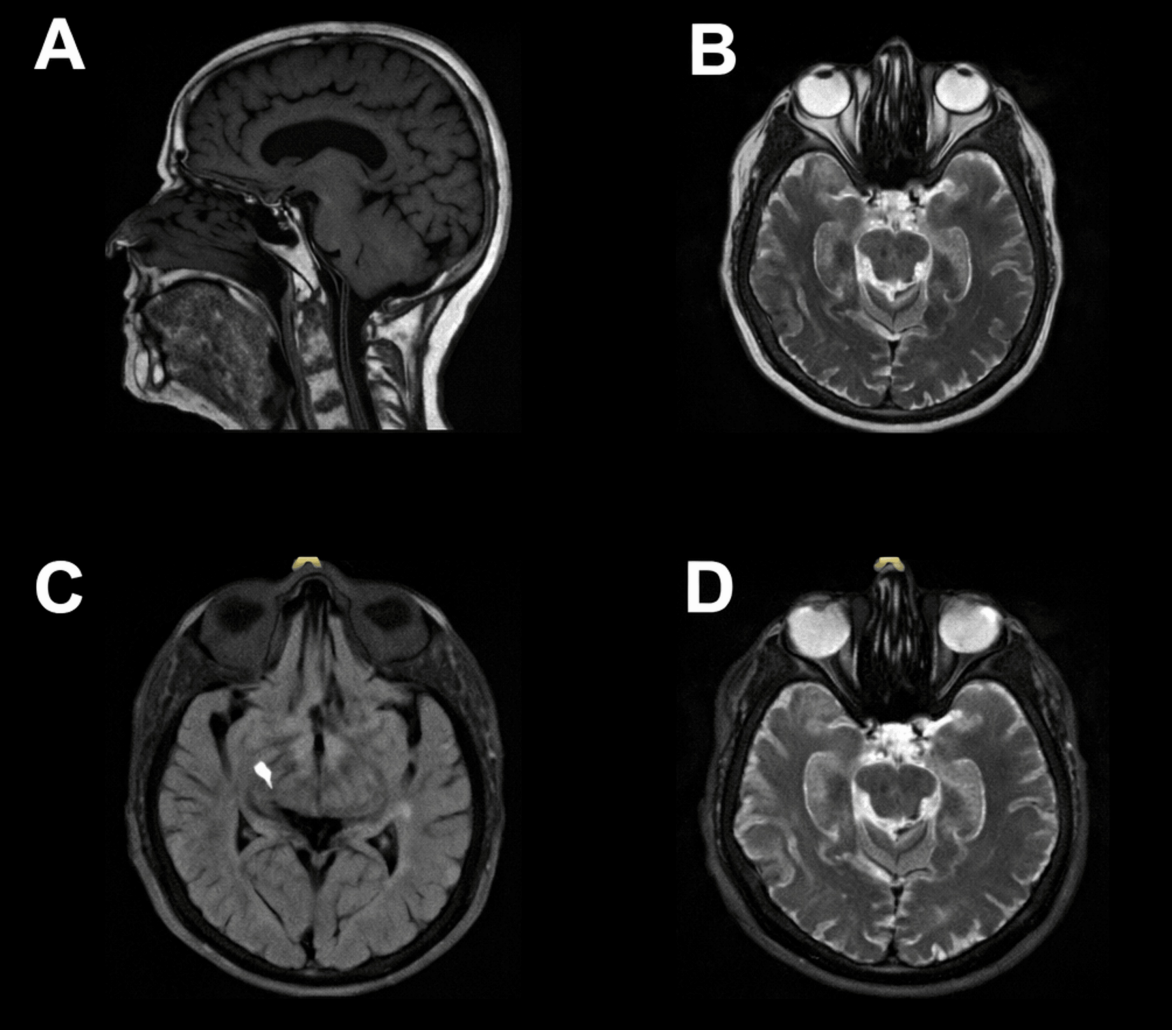
Three-month follow-up MRI demonstrating marked interval reduction of the prior hemorrhagic lesion. A. Sagittal T1-weighted MRI showing normal midline structures with subtle residual signal abnormality at the site of the prior lesion. B. Axial T2-weighted MRI demonstrating symmetric appearance of the midbrain with mild residual hyperintensity in the region of the previous hemorrhage. C. Axial FLAIR sequence showing a small residual focus of signal alteration in the left perimesencephalic region. D. Axial T2-weighted MRI with minimal linear hyperintensity in the area corresponding to prior hemorrhage. FLAIR, fluid-attenuated inversion recovery

A subsequent MRI of the brain obtained three months later demonstrated marked interval reduction in the size of the hemorrhagic lesion, with only a small amount of residual hemosiderin staining and minimal linear enhancement, likely representing granulation tissue (Figure 5). These findings were consistent with progressive resolution of the hemorrhage. Continued radiographic surveillance with interval MRI was recommended to confirm the expected evolution and to definitively exclude an underlying neoplastic process.

## Discussion

NCC is a parasitic infection of the central nervous system caused by *Taenia solium* larvae and remains one of the most prevalent causes of acquired epilepsy worldwide, particularly in Latin America, sub-Saharan Africa, and Southeast Asia. Despite improved public health measures in many endemic regions, NCC continues to be diagnosed in high-income countries due to migration from endemic areas. Colombia remains an important source of cases, with national data showing a decline but persistent incidence of taeniasis and cysticercosis during recent years. Given this epidemiologic context, NCC should remain in the differential diagnosis of intracranial lesions in immigrants from endemic regions [3,5].

Although NCC typically presents with seizures, headaches, or focal deficits, hemorrhagic presentations are exceptionally rare and can closely mimic primary or metastatic neoplasms on neuroimaging. This rarity is clinically significant, as hemorrhagic NCC represents an uncommon manifestation of a common disease, increasing the risk of misdiagnosis and potentially unnecessary invasive interventions [2]. In our patient, the initial CT demonstrated an acute left frontal intraparenchymal hemorrhage without a clear underlying mass, prompting consideration of hypertensive hemorrhage, anticoagulant-related bleeding, vascular malformations, neoplastic etiologies given her remote colon cancer, and, less commonly, parasitic infection. However, several features narrowed the differential. The absence of persistent or nodular enhancement, the presence of an eccentric intracystic nodule more compatible with a scolex, and the lesion’s progressive spontaneous regression on serial MRI argued strongly against primary or metastatic neoplasm. Cavernous malformation and abscess were also unlikely due to the lack of characteristic imaging patterns. While warfarin likely amplified the hemorrhagic component, anticoagulation alone did not explain the cystic morphology. Overall, the radiologic features and longitudinal evolution were most consistent with hemorrhagic NCC.

Hemorrhagic complications of NCC have been described only rarely in the literature. Prior reports have shown that parenchymal cysticercal lesions can occasionally precipitate intracerebral hemorrhage or stroke-like presentations, likely through mechanisms involving local vasculitis, inflammatory vascular injury, and weakening of small intraparenchymal vessels. Other published cases have similarly documented acute hemorrhage in the setting of degenerating cysts, emphasizing that the inflammatory phase of cyst evolution can disrupt vascular integrity and lead to atypical hemorrhagic manifestations. However, these earlier cases occurred in patients who were not receiving anticoagulation [6,7]. A review of hemorrhagic cerebrovascular events associated with NCC further supports this mechanism, noting that degenerating cysts may provoke intense perilesional inflammation and small-vessel injury capable of producing focal hemorrhage, even in the absence of coagulopathy [8]. These findings closely parallel the pathophysiology proposed in our case. In contrast, our patient’s chronic warfarin therapy likely amplified the hemorrhagic component, producing a more dramatic radiologic appearance and contributing to diagnostic uncertainty. The interaction between cyst degeneration, inflammation-induced vascular fragility, and therapeutic anticoagulation provides a plausible explanation for both the hemorrhage and the unique clinical presentation observed in this case.

The pathophysiology underlying hemorrhagic NCC is incompletely understood but is thought to be related to the degenerative (colloidal or granular-nodular) phase of the cyst, during which parasite degeneration triggers an intense host inflammatory response. This inflammatory phase is characterized by disruption of the blood-brain barrier, perilesional vasculitis, and weakening of small intraparenchymal vessels, predisposing to focal hemorrhage. These mechanisms can predispose to parenchymal bleeding, particularly in the presence of coagulopathy [9]. In this case, the patient’s chronic warfarin therapy for a mechanical aortic valve likely amplified the hemorrhagic component. Anticoagulants such as warfarin are well-established contributors to intracranial bleeding and may unmask or exacerbate hemorrhage arising from otherwise indolent lesions. Thus, the hemorrhagic pattern of NCC in this patient may have resulted from the combination of an inflamed, degenerating cyst and therapeutic anticoagulation.

Advanced neuroimaging played a central role in diagnosis. MRI revealed a hemorrhagic cystic lesion with an internal T2-hyperintense component and a nodular focus compatible with a scolex, an essential diagnostic clue distinguishing NCC from neoplasia or abscess [10]. The lesion showed minimal enhancement and surrounding vasogenic edema, features supporting an infectious-inflammatory process. Also, Western blot testing for *Taenia solium* antibodies was negative, underscoring that the diagnosis relied chiefly on radiologic features and the overall clinical scenario. This finding aligns with prior studies showing sensitivities of only 50% in patients with solitary parenchymal lesions, thereby limiting the reliability of serologic testing as an independent diagnostic tool in this setting [11]. Therefore, in this clinical context, diagnosis relied primarily on the integration of epidemiologic exposure, characteristic imaging features, and radiologic evolution rather than serologic confirmation. A stereotactic biopsy was not pursued because the lesion demonstrated no nodular or mass-like enhancement, showed an eccentric intracystic nodule highly compatible with a scolex, and exhibited progressive spontaneous radiologic regression on serial MRI, features strongly favoring degenerating NCC over neoplastic disease. The patient’s history of colon cancer was remote, without evidence of systemic malignancy. Additionally, solitary parenchymal NCC is known to produce false-negative serology, making imaging evolution more diagnostically reliable than antibody testing in this context. Given these factors, the multidisciplinary team concluded that a biopsy would pose unnecessary risk without altering management.

Although serological and histological confirmation was not available, NCC is frequently diagnosed based on clinical-radiologic criteria when biopsy is not feasible. As summarized by Guzmán and García-HH in their review of the current diagnostic criteria, a diagnosis of probable NCC can be made when typical neuroimaging features, expected lesion evolution, epidemiologic likelihood, and exclusion of more plausible alternative diagnoses are present [12]. In our patient, the imaging characteristics, their interval evolution, and the epidemiologic context fulfill these criteria despite negative serology, which is known to occur in solitary or degenerating lesions. Importantly, the lesion demonstrated radiologic improvement after antiparasitic therapy, a therapeutic response that further supports the diagnosis of NCC rather than a primary hematologic or vascular etiology. Additionally, the hemorrhagic component cannot be fully attributed to anticoagulation alone. The bleeding was centered on a pre-existing structural lesion rather than occurring in a typical hypertensive or anticoagulant-related location, and the patient had no history of severe hypertension. The pattern of edema, the morphology of the lesion, and its radiologic evolution were more consistent with a degenerating parasitic cyst than with a primary anticoagulant-associated intracerebral hemorrhage. This suggests that the underlying lesion is predisposed to hemorrhage, with anticoagulation acting as a contributing factor rather than the sole mechanism.

A secondary but clinically relevant aspect of the patient’s presentation was severe hyponatremia due to SIADH, which is a recognized complication of intracranial processes, including infections, hemorrhage, and neoplasms, and can independently contribute to encephalopathy. In this patient, hyponatremia likely compounded her altered mental status. Management with hypertonic saline and fluid restriction led to clinical improvement, underscoring the importance of promptly addressing metabolic derangements in neurocritical care [13,14].

Management of anticoagulant-associated intracranial hemorrhage (AAICH) posed a significant challenge. Given the risk of hematoma expansion in the setting of warfarin therapy, rapid reversal using four-factor PCC was appropriate and aligned with current guidelines for vitamin K antagonist-associated intracranial hemorrhage. Because the patient’s hemorrhage was attributed to an underlying inflammatory-infectious lesion rather than uncontrolled anticoagulation, temporary cessation of warfarin was necessary while balancing the thromboembolic risk associated with her mechanical valve. Such complex scenarios necessitate individualized, multidisciplinary decision-making to determine the optimal timing of anticoagulation resumption [15]. Given the high thromboembolic risk associated with mechanical aortic valves, re-initiation of warfarin following radiographic stability was clinically necessary and aligned with current AAICH guideline recommendations.

Therapeutic management in cases of hemorrhagic NCC requires coordinated attention to both the underlying parasitic infection and the acute neurological complications. Albendazole is considered the preferred antiparasitic agent for active parenchymal disease due to its superior cerebrospinal fluid penetration and greater cysticidal efficacy compared with praziquantel [2]. Typical dosing consists of 15 mg/kg/day for seven to 14 days. Concomitant corticosteroid therapy, such as dexamethasone (0.1 mg/kg/day) or prednisone (1 mg/kg/day), is essential to mitigate the inflammatory response triggered by cyst degeneration, thereby reducing perilesional edema and the risk of neurological deterioration. In hemorrhagic presentations, corticosteroids are particularly important to limit secondary injury [3].

The patient’s clinical and radiologic course further supported the diagnosis of NCC. Serial MRIs at one and three months demonstrated progressive reduction in lesion size and resolution of perilesional edema, consistent with healing from an inflammatory hemorrhagic lesion. Minimal linear enhancement observed on later imaging was compatible with granulation tissue rather than neoplastic progression. This pattern of radiologic resolution highlights the critical role of serial imaging in confirming the diagnosis, monitoring treatment response, and avoiding unnecessary brain biopsy or surgical intervention [16].

## Conclusions

This case describes a rare hemorrhagic presentation of parenchymal NCC in an elderly patient on chronic anticoagulation. Diagnosis relied primarily on epidemiologic risk factors and characteristic MRI findings, particularly the presence of an eccentric scolex and progressive radiologic regression, given the low sensitivity of serologic testing in solitary lesions. Anticoagulation likely amplified hemorrhage from a degenerating cyst, creating a challenging clinical and radiologic scenario. Although histopathologic confirmation was not obtained, the radiologic evolution and therapeutic response supported a presumptive diagnosis consistent with established criteria for solitary parenchymal NCC.

The case also highlights the importance of recognizing systemic complications such as SIADH, which contributed to the patient’s encephalopathy and required timely correction. Effective management required a multidisciplinary approach, balancing anticoagulation reversal, metabolic stabilization, and initiation of antiparasitic therapy. In patients with mechanical heart valves, coordinated decision-making among neurology, neurosurgery, and cardiology is essential to safely resume anticoagulation once radiographic stability is achieved. Ultimately, serial neuroimaging proved essential in confirming the diagnosis, guiding non-invasive management, and avoiding unnecessary procedures such as brain biopsy.
